# Supplementary material for: Wastewater-based epidemiology predicts COVID-19-induced weekly new hospital admissions in over 150 USA counties
Source: Nat Commun. 2023 Jul 28;14:4548. doi: 10.1038/s41467-023-40305-x (PMC10382499; doi:10.1038/s41467-023-40305-x)
Supplement: Supplementary file 1 — Supplementary Information [file 41467_2023_40305_MOESM1_ESM.pdf]

Wastewater-based epidemiology predicts COVID-19-induced weekly new hospital admissions in over 150 USA counties

Xuan Li [a], Huan Liu [a], Li Gao [b], Samendra P. Sherchan [c,d], Ting Zhou [a], Stuart J. Khan [e], Mark C.M. van Loosdrecht [f], Qilin Wang [a,*]

[a] Centre for Technology in Water and Wastewater, School of Civil and Environmental Engineering, University of Technology Sydney, Ultimo, NSW, 2007, Australia.

[b] South East Water, 101 Wells Street, Frankston, VIC 3199, Australia

[c] Department of Biology, Morgan State University, Baltimore, MD, USA.

[d] Department of Environmental Health Sciences, School of Public Health and Tropical Medicine, Tulane University, New Orleans, LA, USA.

[e] Water Research Centre, School of Civil and Environmental Engineering, University of New South Wales, Sydney, NSW 2052, Australia

[f] Department of Biotechnology, Delft University of Technology, Julianalaan 67, 2628 BC Delft, the Netherlands.

*Corresponding author:

E-mail: Qilin.Wang@uts.edu.au

Tables

Table S1. Counties involved, with their state and population size used for the modeling of COVID-19-induced hospitalization indicators (99 counties in total).

FIPS code	State	County	Population size [a]
1089	AL	Madison County, AL	372909
4019	AZ	Pima County, AZ	1047279
5007	AR	Benton County, AR	279141
5119	AR	Pulaski County, AR	391911
6053	CA	Monterey County, CA	434061
6061	CA	Placer County, CA	398329
6065	CA	Riverside County, CA	2470546
6067	CA	Sacramento County, CA	1552058
6073	CA	San Diego County, CA	3338330
6087	CA	Santa Cruz County, CA	273213
6097	CA	Sonoma County, CA	494336
8005	CO	Arapahoe County, CO	656590
9001	CT	Fairfield County, CT	943332
9003	CT	Hartford County, CT	891720
9007	CT	Middlesex County, CT	162436
9009	CT	New Haven County, CT	854757
10003	DE	New Castle County, DE	558753
12009	FL	Brevard County, FL	601942
12057	FL	Hillsborough County, FL	1471968
12086	FL	Miami-Dade County, FL	2716940
12095	FL	Orange County, FL	1393452
12099	FL	Palm Beach County, FL	1496770
12117	FL	Seminole County, FL	471826
16001	ID	Ada County, ID	481587
16057	ID	Latah County, ID	40108
17091	IL	Kankakee County, IL	109862
17143	IL	Peoria County, IL	179179
17201	IL	Winnebago County, IL	282572
18057	IN	Hamilton County, IN	338011
18089	IN	Lake County, IN	485493
18157	IN	Tippecanoe County, IN	195732

19153	IA	Polk County, IA	490161
20091	KS	Johnson County, KS	602401
20173	KS	Sedgwick County, KS	516042
21097	KY	Harrison County, KY	18886
21111	KY	Jefferson County, KY	766757
21117	KY	Kenton County, KY	166998
22033	LA	East Baton Rouge Parish, LA	440059
23001	ME	Androscoggin County, ME	108277
23003	ME	Aroostook County, ME	67055
23005	ME	Cumberland County, ME	295003
23011	ME	Kennebec County, ME	122302
23019	ME	Penobscot County, ME	152148
23027	ME	Waldo County, ME	39715
23031	ME	York County, ME	207641
24037	MD	St. Mary's County, MD	113510
25003	MA	Berkshire County, MA	124944
25005	MA	Bristol County, MA	565217
25009	MA	Essex County, MA	789034
25015	MA	Hampshire County, MA	160830
25017	MA	Middlesex County, MA	1611699
25023	MA	Plymouth County, MA	521202
25025	MA	Suffolk County, MA	803907
25027	MA	Worcester County, MA	830622
27037	MN	Dakota County, MN	429021
27053	MN	Hennepin County, MN	1265843
28049	MS	Hinds County, MS	231840
29031	MO	Cape Girardeau County, MO	78871
32003	NV	Clark County, NV	2266715
32007	NV	Elko County, NV	52778
32031	NV	Washoe County, NV	471519
33013	NH	Merrimack County, NH	151391
34013	NJ	Essex County, NJ	798975
34023	NJ	Middlesex County, NJ	825062
34025	NJ	Monmouth County, NJ	618795
34039	NJ	Union County, NJ	556341
36059	NY	Nassau County, NY	1356924

37051	NC	Cumberland County, NC	335509
39153	OH	Summit County, OH	541013
40109	OK	Oklahoma County, OK	797434
41017	OR	Deschutes County, OR	197692
42029	PA	Chester County, PA	524989
42043	PA	Dauphin County, PA	278299
42049	PA	Erie County, PA	269728
42055	PA	Franklin County, PA	155027
42063	PA	Indiana County, PA	84073
42069	PA	Lackawanna County, PA	209674
42079	PA	Luzerne County, PA	317417
42091	PA	Montgomery County, PA	830915
42129	PA	Westmoreland County, PA	348899
44007	RI	Providence County, RI	638931
44009	RI	Washington County, RI	125577
45045	SC	Greenville County, SC	523542
47065	TN	Hamilton County, TN	367804
47163	TN	Sullivan County, TN	158348
48257	TX	Kaufman County, TX	136154
48453	TX	Travis County, TX	1273954
49011	UT	Davis County, UT	355481
49035	UT	Salt Lake County, UT	1160437
50007	VT	Chittenden County, VT	163774
51013	VA	Arlington County, VA	236842
51107	VA	Loudoun County, VA	413538
51179	VA	Stafford County, VA	152882
51510	VA	Alexandria City, VA	159428
51660	VA	Harrisonburg City, VA	53016
53005	WA	Benton County, WA	204390
53057	WA	Skagit County, WA	129205
54069	WV	Ohio County, WV	41411
55073	WI	Marathon County, WI	135692

[a]: Based on the census data in 2019.

Table S2. Model performance during model establishment stage June 2021-May 2022

Indicators	Model	Hos1w			Hos2w			Hos3w			Hos4w		
		R	MAE	NMAE	R	MAE	NMAE	R	MAE	NMAE	R	MAE	NMAE
Weekly new admission	WBE	0.91	2.87	0.22	0.90	2.77	0.22	0.92	3.15	0.24	0.90	3.48	0.27
	Record	0.86	3.18	0.25	0.87	4.85	0.38	0.83	5.40	0.42	0.80	5.57	0.43
	Case	0.86	3.18	0.25	0.87	4.39	0.34	0.85	4.88	0.38	0.81	5.37	0.41
Census inpatient sum	WBE	0.89	34.81	0.30	0.89	32.23	0.29	0.96	31.18	0.28	0.93	24.87	0.30
	Record	0.93	28.38	0.25	0.89	35.42	0.32	0.86	39.12	0.35	0.94	27.33	0.34
	Case	0.89	31.39	0.28	0.88	36.26	0.34	0.86	35.57	0.32	0.95	23.72	0.31
Census inpatient average	WBE	0.95	3.97	0.26	0.97	3.43	0.22	0.95	3.54	0.23	0.94	3.76	0.24
	Record	0.96	3.49	0.23	0.91	5.03	0.33	0.88	5.84	0.38	0.86	6.24	0.40
	Case	0.91	2.87	0.22	0.90	2.77	0.22	0.92	3.15	0.24	0.90	3.48	0.27

Note: Hos1w, Hos2w, Hos3w, and Hos4w represent the first, second, third, and fourth week after wastewater sampling, respectively. R denotes the correlation coefficient, MAE indicates the mean absolute error, and NMAE refers to the normalized mean absolute error (refer to the Methods section for detailed calculations). WBE means the wastewater-based epidemiology.

Table S3 Performance of models using WBE with selective explanatory factors

		Hos1w			Hos2w			Hos3w			Hos4w		
		R	MAE	NMAE	R	MAE	NMAE	R	MAE	NMAE	R	MAE	NMAE
Weekly new admission	WBE	0.73	2.93	0.28	0.76	2.91	0.28	0.74	2.89	0.28	0.71	2.89	0.29

Note: Hos1w, Hos2w, Hos3w, and Hos4w represent the first, second, third, and fourth week after wastewater sampling, respectively. R denotes the correlation coefficient, MAE indicates the mean absolute error, and NMAE refers to the normalized mean absolute error (refer to the Methods section for detailed calculations). WBE means the wastewater-based epidemiology.

Table S4. The information about the 60 counties used for evaluating the model transferability

**FIPS code**	**State**	**County**	**Population size [a]**
1033	AL	Colbert County, AL	55241
4025	AZ	Yavapai County, AZ	235099
5145	AR	White County, AR	78753
6001	CA	Alameda County, CA	1671329
6013	CA	Contra Costa County, CA	1153526
6023	CA	Humboldt County, CA	135558
6037	CA	Los Angeles County, CA	10039107
9011	CT	New London County, CT	265206
11001	DC	Washington, DC	705749
12001	FL	Alachua County, FL	269043
12103	FL	Pinellas County, FL	974996
13245	GA	Richmond County, GA	202518
17031	IL	Cook County, IL	5150233
18003	IN	Allen County, IN	379299
18005	IN	Bartholomew County, IN	83779
18035	IN	Delaware County, IN	114135
18037	IN	Dubois County, IN	42736
18105	IN	Monroe County, IN	148431
18141	IN	St. Joseph County, IN	271826
18177	IN	Wayne County, IN	65884
19103	IA	Johnson County, IA	151140
19193	IA	Woodbury County, IA	103107
20155	KS	Reno County, KS	61998
20177	KS	Shawnee County, KS	176875
22051	LA	Jefferson Parish, LA	432493
22055	LA	Lafayette Parish, LA	244390
24001	MD	Allegany County, MD	70416
24003	MD	Anne Arundel County, MD	579234
24027	MD	Howard County, MD	325690
24043	MD	Washington County, MD	151049
25013	MA	Hampden County, MA	466372
34003	NJ	Bergen County, NJ	932202
34017	NJ	Hudson County, NJ	672391
34031	NJ	Passaic County, NJ	501826
35001	NM	Bernalillo County, NM	679121
35049	NM	Santa Fe County, NM	150358
38035	ND	Grand Forks County, ND	69451
40019	OK	Carter County, OK	48111
40031	OK	Comanche County, OK	120749
41005	OR	Clackamas County, OR	418187
41051	OR	Multnomah County, OR	812855
42017	PA	Bucks County, PA	628270
42019	PA	Butler County, PA	187853

44003	RI	Kent County, RI	164292
45041	SC	Florence County, SC	138293
45047	SC	Greenwood County, SC	70811
45051	SC	Horry County, SC	354081
46099	SD	Minnehaha County, SD	193134
46103	SD	Pennington County, SD	113775
47037	TN	Davidson County, TN	694144
48113	TX	Dallas County, TX	2635516
48141	TX	El Paso County, TX	839238
48439	TX	Tarrant County, TX	2102515
50003	VT	Bennington County, VT	35470
51085	VA	Hanover County, VA	107766
51195	VA	Wise County, VA	37383
51730	VA	Petersburg city, VA	31346
53011	WA	Clark County, WA	488241
53061	WA	Snohomish County, WA	822083
55025	WI	Dane County, WI	546695

[a]: Based on the census data in 2019.

Table S5. Model performance using non-transformed data and Box-Cox transformed data.

	**Non-transformed data**				**Transformed data**			
	Training	test	validation	all	Training	test	validation	all
**R**	0.98	0.87	0.88	0.95	0.97	0.86	0.87	0.94
**MAE**	2.80	6.35	5.52	3.74	2.81	6.38	5.34	3.73
**NMAE**	0.18	0.38	0.37	0.24	0.18	0.38	0.36	0.24

Note: R denotes the correlation coefficient, MAE indicates the mean absolute error, and NMAE refers to the normalized mean absolute error (refer to the Methods section for detailed calculations).

Table S6. *p*-values for the correlation in Figure 3a

	Weekly new admission (Hos_wn)				Census inpatient sum (Hos_cs)				Census inpatient average (Hos_ca)			
	Hos1w	hos2w	Hos3w	Hos4w	Hos1w	hos2w	Hos3w	Hos4w	Hos1w	hos2w	Hos3w	Hos4w
C_{RNA}	1.81E-225	1.03E-234	1.32E-212	1.19E-163	2.59E-242	9.17E-253	1.12E-226	9.01E-175	2.02E-321	4.52E-306	1.27E-250	1.13E-173
Vaccine_1st	8.05E-06	4.85E-12	8.24E-22	1.37E-34	2.80E-06	6.88E-13	5.30E-23	6.33E-36	2.10E-03	1.74E-08	1.33E-16	1.77E-27
Vaccine_2nd	6.02E-09	4.85E-16	1.69E-26	1.99E-39	1.30E-08	1.03E-15	4.69E-26	1.63E-38	3.04E-06	1.99E-12	2.04E-21	2.01E-32
Population	2.53E-10	5.30E-11	9.73E-11	2.26E-10	3.16E-04	1.25E-04	9.56E-05	1.26E-04	1.74E-08	4.36E-09	1.59E-08	2.02E-08
Overall_VI	1.18E-50	2.88E-51	1.01E-51	1.06E-50	3.09E-45	2.02E-46	2.09E-47	1.29E-46	1.05E-36	7.30E-37	1.86E-36	1.16E-36
Socioeconomic status	2.81E-25	6.92E-25	1.84E-24	1.28E-23	1.33E-26	1.54E-26	2.90E-26	2.50E-25	8.86E-20	7.60E-20	3.86E-19	3.06E-19
Minority	1.00E-02	6.65E-03	7.17E-03	7.65E-03	9.19E-01	9.55E-01	8.77E-01	9.06E-01	4.24E-06	1.14E-06	1.91E-06	1.91E-06
Household transportation	2.77E-24	4.89E-23	2.73E-22	1.15E-20	8.99E-32	5.13E-31	3.15E-30	8.41E-29	1.31E-21	9.75E-21	6.33E-20	2.33E-19
Epidemiological	1.18E-31	1.81E-31	8.27E-31	1.02E-28	3.86E-44	1.15E-44	1.54E-44	7.61E-43	1.34E-13	4.92E-13	2.16E-12	2.08E-11
Healthcare system	6.04E-02	7.55E-02	9.84E-02	1.23E-01	2.67E-04	2.63E-04	3.49E-04	2.78E-04	4.93E-01	5.46E-01	5.25E-01	6.04E-01
High risk environment	2.84E-37	3.39E-37	7.39E-38	2.80E-38	9.08E-41	3.08E-41	4.59E-42	1.34E-42	1.83E-16	2.68E-15	1.20E-15	3.29E-16
Population density	9.61E-08	1.65E-08	1.34E-08	1.11E-08	1.86E-03	5.88E-04	3.12E-04	2.52E-04	3.31E-09	2.65E-10	6.11E-11	1.61E-11
Precipitation	4.66E-02	5.03E-01	4.46E-01	2.45E-01	2.95E-01	2.36E-01	1.36E-01	3.61E-02	6.23E-01	6.89E-01	6.75E-01	1.84E-01
T_a	4.19E-04	2.61E-01	1.62E-08	1.44E-22	8.69E-07	7.42E-01	1.94E-05	2.40E-17	1.08E-02	2.94E-02	3.09E-11	1.15E-25
T_w	4.10E-04	2.63E-01	1.68E-08	1.54E-22	8.44E-07	7.37E-01	2.00E-05	2.54E-17	1.08E-02	2.94E-02	3.10E-11	1.17E-25

Table S7. *p*-values for the correlation in Figure S8

	Weekly new admission (Hos_wn)				Census inpatient sum (Hos_cs)				Census inpatient average (Hos_ca)			
	Hos1w	hos2w	Hos3w	Hos4w	Hos1w	hos2w	Hos3w	Hos4w	Hos1w	hos2w	Hos3w	Hos4w
Weekly new cases	0.00	3.68E-308	4.87E-233	1.18E-148	0.00	0.00	1.88E-242	5.29E-155	0.00	0.00	4.74E-226	6.13E-132
Potive_rate	1.64E-155	8.96E-212	9.00E-240	1.92E-221	2.75E-165	7.47E-223	9.21E-250	8.54E-232	7.01E-226	9.52E-278	2.57E-279	1.78E-234
Hos0w	0.00	0.00	0.00	0.00	0.00E	0.00	0.00	1.43E-273	0.00	5.18E-302	8.97E-179	1.46E-100
Overall_VI	1.18E-50	2.88E-51	1.01E-51	1.06E-50	3.09E-45	2.02E-46	2.09E-47	1.29E-46	1.05E-36	7.30E-37	1.86E-36	1.16E-36
Socioeconomic status	2.81E-25	6.92E-25	1.84E-24	1.28E-23	1.33E-26	1.54E-26	2.90E-26	2.50E-25	8.86E-20	7.60E-20	3.86E-19	3.06E-19
Minority	1.00E-02	6.65E-03	7.17E-03	7.65E-03	9.19E-01	9.55E-01	8.77E-01	9.06E-01	4.24E-06	1.14E-06	1.91E-06	1.91E-06
Household transportation	2.77E-24	4.89E-23	2.73E-22	1.15E-20	8.99E-32	5.13E-31	3.15E-30	8.41E-29	1.31E-21	9.75E-21	6.33E-20	2.33E-19
Epidemiological	1.18E-31	1.81E-31	8.27E-31	1.02E-28	3.86E-44	1.15E-44	1.54E-44	7.61E-43	1.34E-13	4.92E-13	2.16E-12	2.08E-11
Healthcare system	6.04E-02	7.55E-02	9.84E-02	1.23E-01	2.67E-04	2.63E-04	3.49E-04	2.78E-04	4.93E-01	5.46E-01	5.25E-01	6.04E-01
High risk environment	2.84E-37	3.39E-37	7.39E-38	2.80E-38	9.08E-41	3.08E-41	4.59E-42	1.34E-42	1.83E-16	2.68E-15	1.20E-15	3.29E-16
Population density	9.61E-08	1.65E-08	1.34E-08	1.11E-08	1.86E-03	5.88E-04	3.12E-04	2.52E-04	3.31E-09	2.65E-10	6.11E-11	1.61E-11
Vaccine_1st	8.05E-06	4.85E-12	8.24E-22	1.37E-34	2.80E-06	6.88E-13	5.30E-23	6.33E-36	2.10E-03	1.74E-08	1.33E-16	1.77E-27
Vaccine_2nd	6.02E-09	4.85E-16	1.69E-26	1.99E-39	1.30E-08	1.03E-15	4.69E-26	1.63E-38	3.04E-06	1.99E-12	2.04E-21	2.01E-32
Population	2.53E-10	5.30E-11	9.73E-11	2.26E-10	3.16E-04	1.25E-04	9.56E-05	1.26E-04	1.74E-08	4.36E-09	1.59E-08	2.02E-08
Precipitation	4.66E-02	5.03E-01	4.46E-01	2.45E-01	2.95E-01	2.36E-01	1.36E-01	3.61E-02	6.23E-01	6.89E-01	6.75E-01	1.84E-01

T_a	4.19E-04	2.61E-01	1.62E-08	1.44E-22	8.69E-07	7.42E-01	1.94E-05	2.40E-17	1.08E-02	2.94E-02	3.09E-11	1.15E-25

Figures

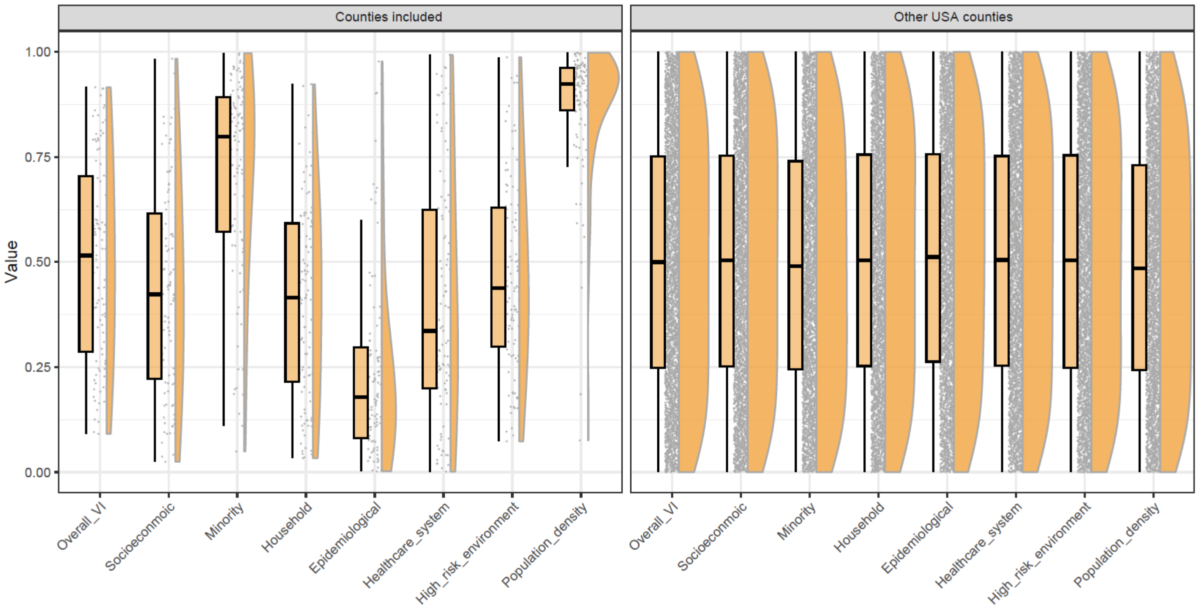

Figure S1. The CCVI of the counties involved and other counties in the USA. The CCVI distribution is represented by a box plot (left), individual points (middle), and a density plot (right) for each index. In box plot, the colored box indicates the 25th and 75th percentiles, and the whiskers indicate the highest and the lowest values of the results. The line in the box indicates median. N=99 for the figure on the left (Counties included) and N=3043 for the figure on the right (Other USA counties).

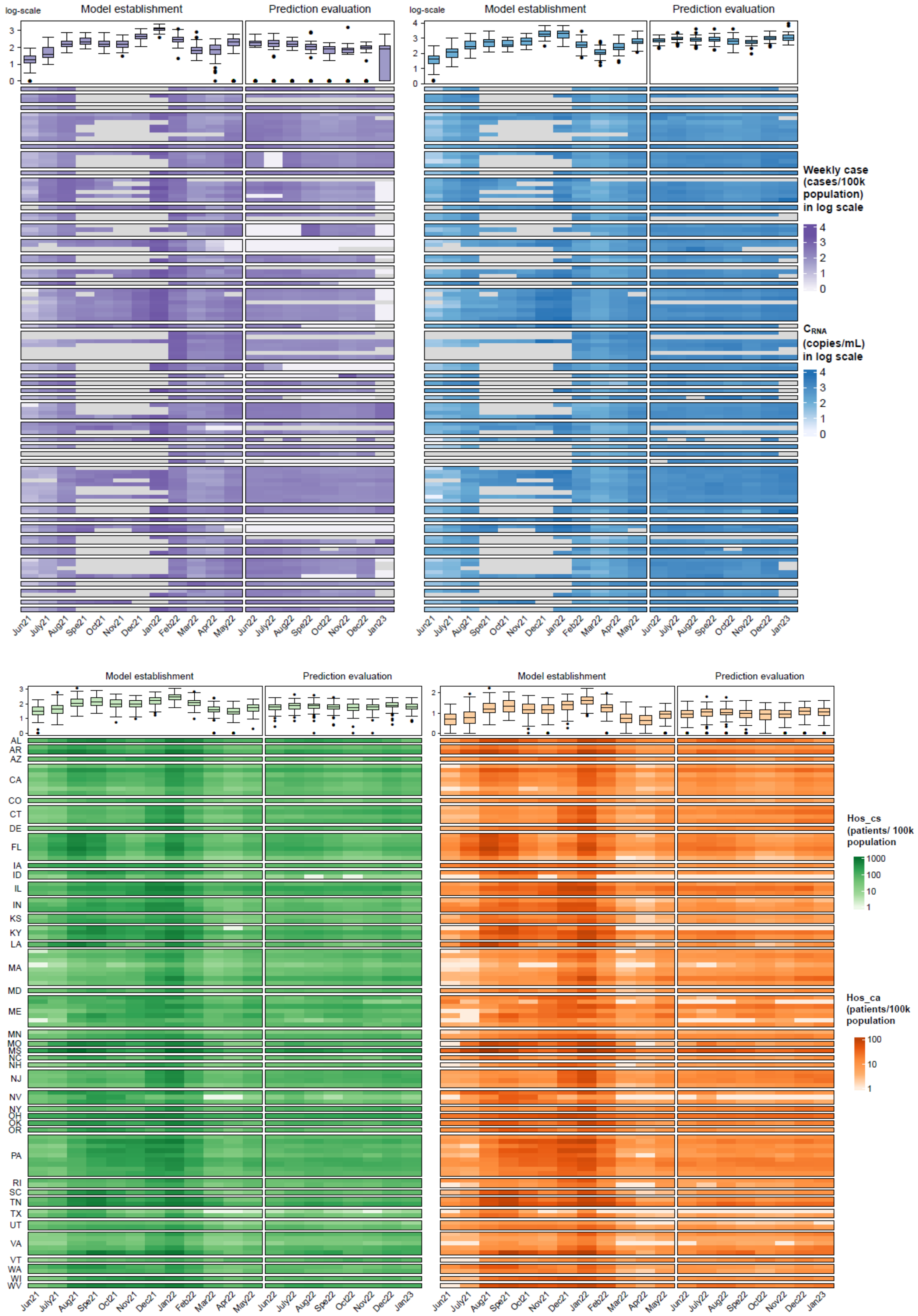

log-scale
Model establishment
Prediction evaluation
Weekly case (cases/100k population) in log scale
C_{RNA} (copies/mL) in log scale
Hos_cs (patients/ 100k population
Hos_ca (patients/100k population
Jun21
July21
Aug21
Spe21
Oct21
Nov21
Dec21
Jan22
Feb22
Mar22
Apr22
May22
Jun22
July22
Aug22
Spe22
Oct22
Nov22
Dec22
Jan23
AL
AR
AZ
CA
CO
CT
DE
FL
IA
ID
IL
IN
KS
KY
LA
MA
MD
ME
MN
MO
MS
NC
NH
NJ
NV
NY
OH
OK
OR
PA
RI
SC
TN
TX
UT
VA
VT
WA
WI
WV

Figure S2. The weekly new COVID-19 cases (cases/100k population), C_{RNA} in wastewater samples, Hos_cs (total number of patients stayed in an inpatient bed during the week), and Hos_ca (daily average number of patients stayed in an inpatient bed during the week), in each county during the study period. The grey cells in the heatmap indicate missing values. The color gradient in each cell represents the monthly average of each indicator. In box plot (top of each subplot), the colored box indicates the 25th and 75th percentiles, and the whiskers indicate the highest and the lowest values of the results. The line in the box indicates median. N=99 for each box.

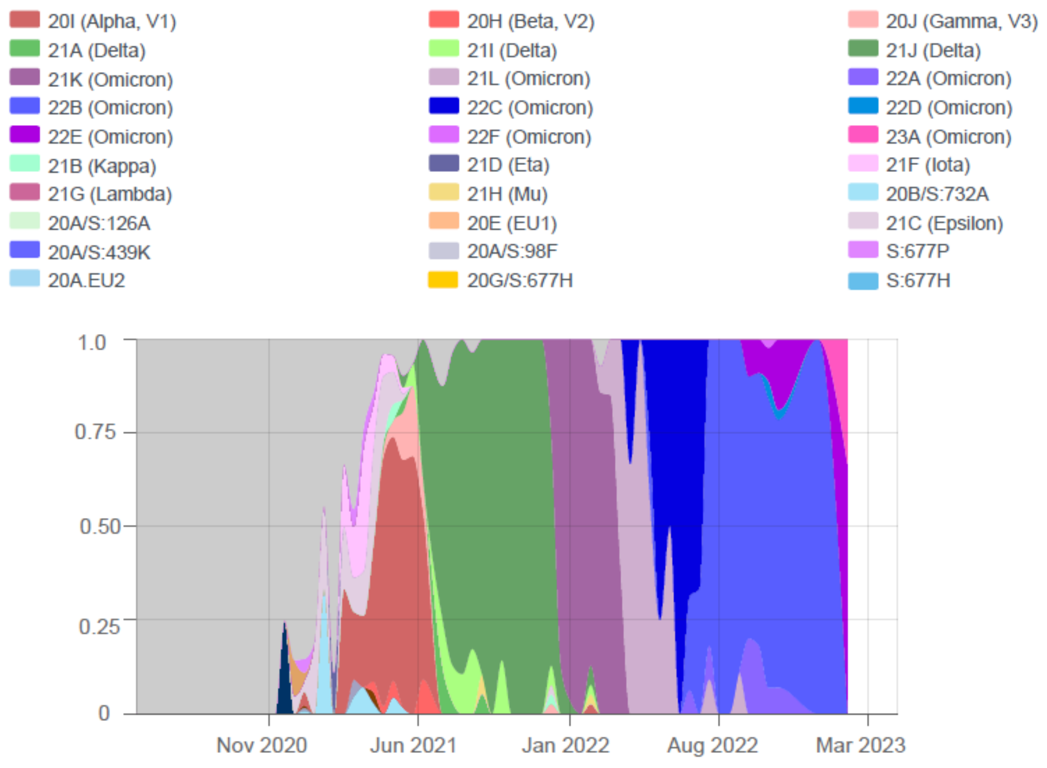

Figure S3. Weekly distribution of the different SARS-CoV-2 variants and subvariants in USA from November 2020 to February 2023 labelled in colors, adapted from Hodcroft [2].

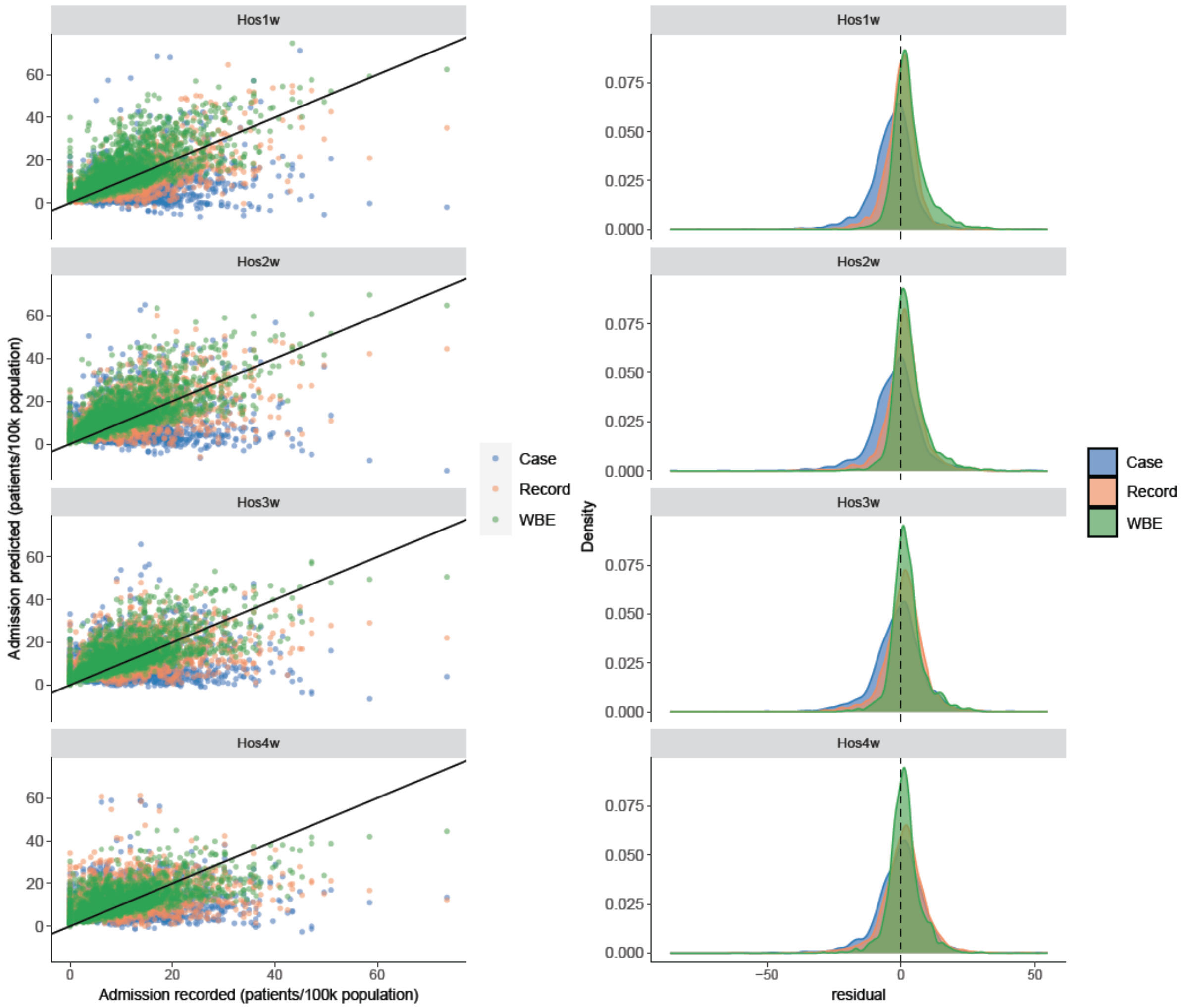

Figure S4. Batch model performance for predicting the future weekly new admission through WBE-based, record-based and case-based models. Hos1w, Hos2w, Hos3w, and Hos4w represent the first, second, third, and fourth week after wastewater sampling, respectively.

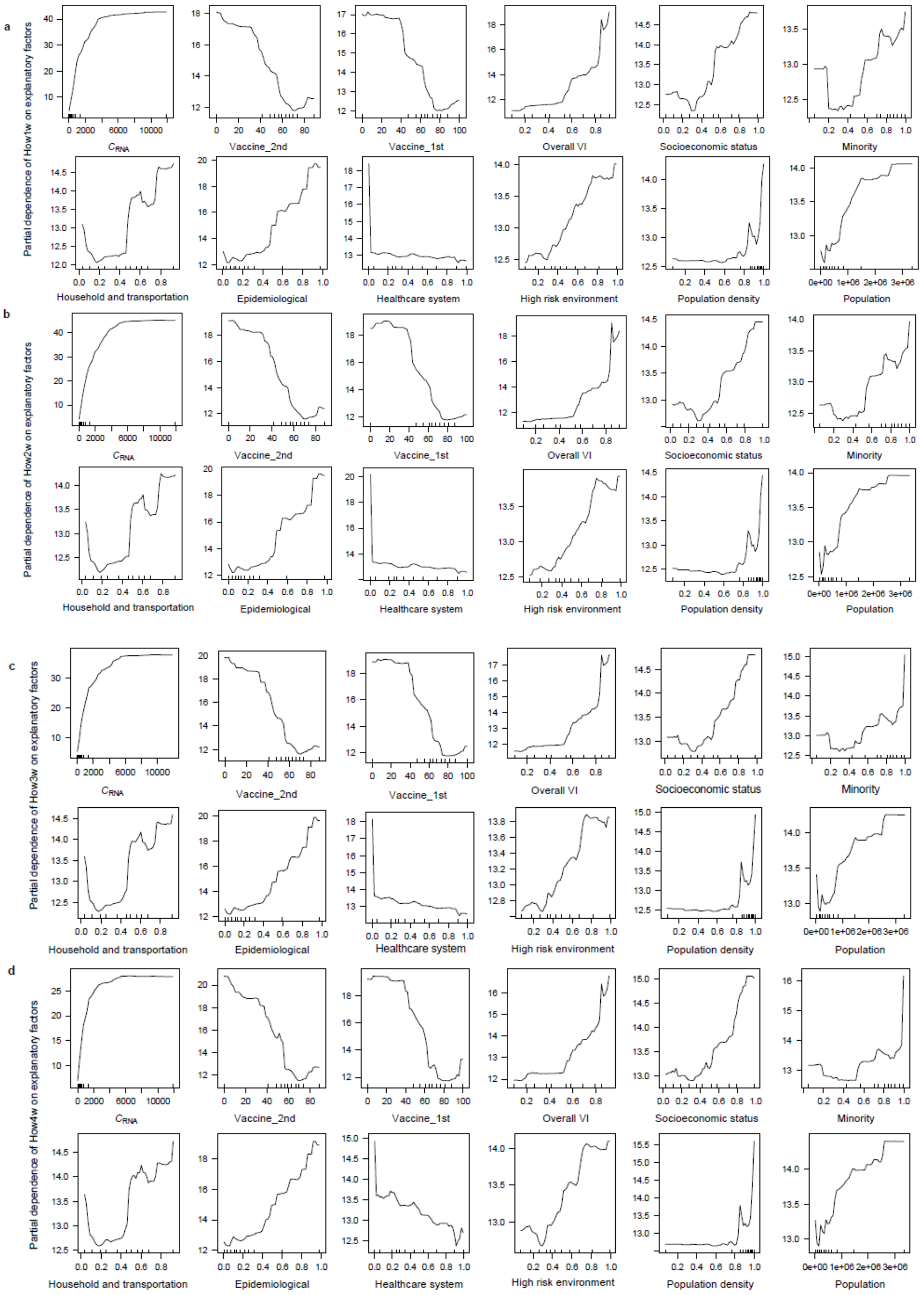

Figure S5. One-factor partial dependence plot of models for predicting Hos_wn with a leading time of Hos1w (subfigure a), Hos2w (subfigure b), Hos3w (subfigure c) and Hos4w (subfigure

d) on the explanatory factors. The tick marks on the x-axis reflected the data density. Hos1w, Hos2w, Hos3w, and Hos4w represent the first, second, third, and fourth week after wastewater sampling, respectively.

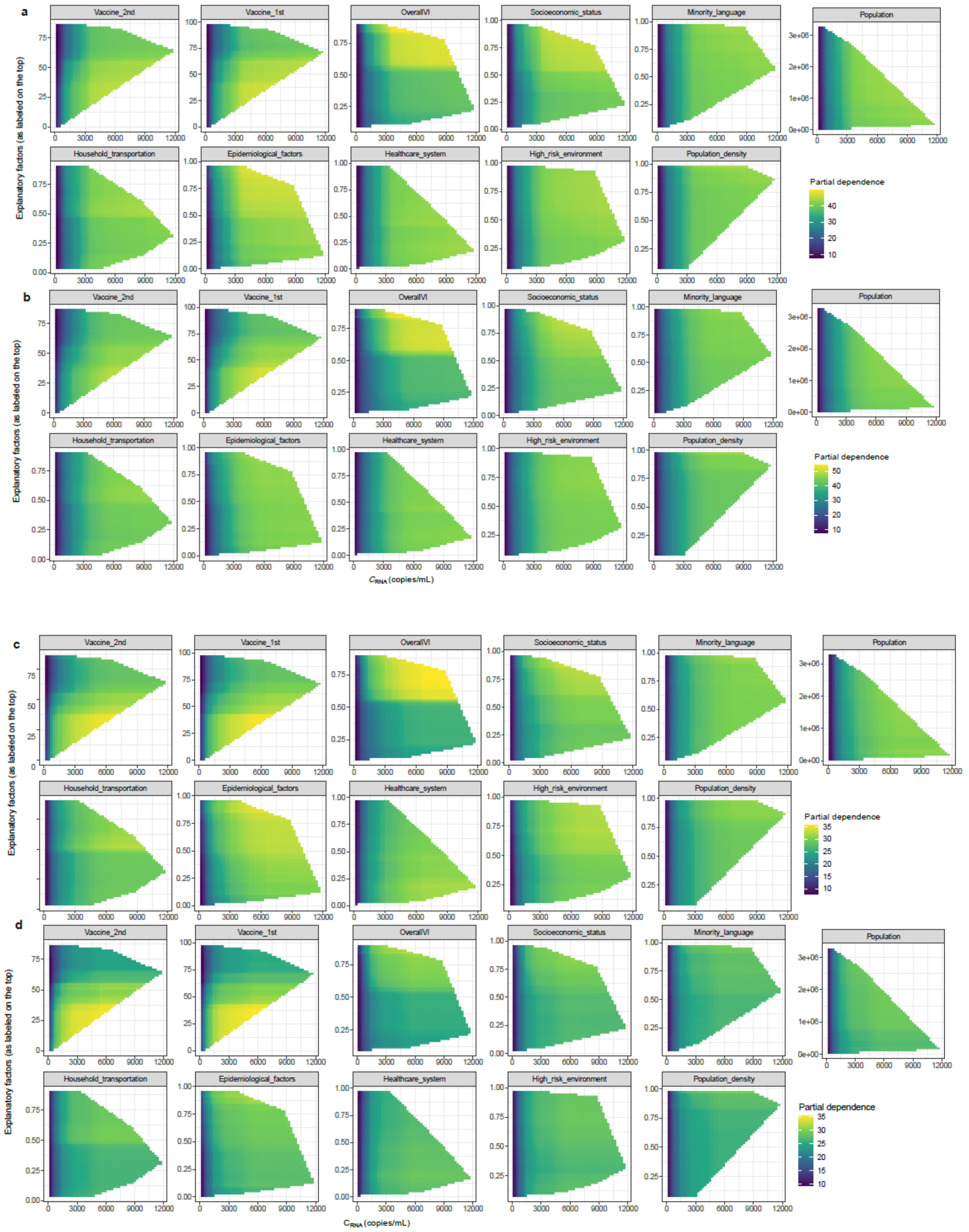

Figure S6. Two-factor partial dependence of models for predicting Hos_wn with a leading time of Hos1w (subfigure a), Hos2w (subfigure b), Hos3w (subfigure c) and Hos4w (subfigure d) on the C_{RNA} and explanatory factors. Hos1w, Hos2w, Hos3w, and Hos4w represent the first, second, third, and fourth week after wastewater sampling, respectively.

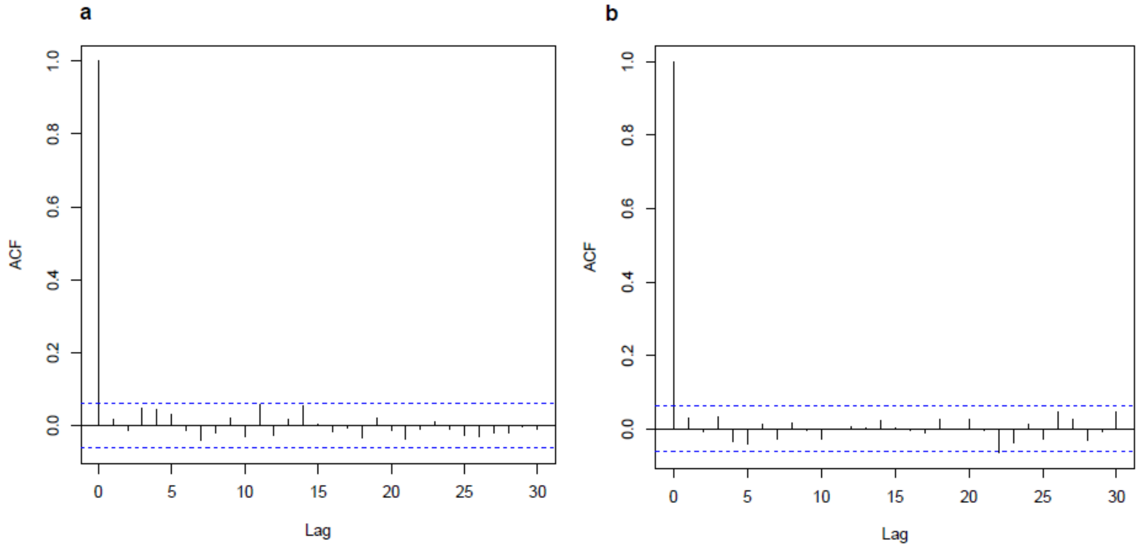

Figure S7. Autocorrelation functions (ACF) plot for the residuals from the batch model (a) and progressively learning model (b) for Weekly new admission (Hos_wn).

Text 1 Wastewater analytical protocols in Biobot

Two protocols were used for the wastewater process. Before February 2022, for each wastewater sample, 15 mL of wastewater were concentrated using Amicon Ultra-15 centrifugal ultrafiltration units (Millipore UFC903096). The viral particles in the concentrate were lysed using viral lysis (AVL) buffer containing carrier RNA (Qiagen 19073) and incubated in a 96-well 2 mL block for more than 10 min. The lysate was adjusted using 100% ethanol for suitable binding conditions before being transferred into RNeasy Mini columns or RNeasy 96 cassettes (Qiagen 74106 or 74181) as per the manufacturer's instructions and eventually eluted in a total of 75 μL of nuclease-free water. After February 2022, viral particles were captured using Ceres NanoTrap particles (SKU 44202) from 9.6 mL of wastewater and lysed with MagMAX Lysis Solution (Applied Biosystems A52606), and 400μL of the resulting lysate is used for nucleic acid isolation with MagMAX DNA/RNA binding beads, as per manufacturer's instructions. The change of wastewater process protocol was confirmed not to introduce a systematic bias by evaluating the sample-to-sample variability [1]. After the wastewater process, 3 μL per reaction of eluted RNA sample was subjected to one-step reverse transcription-quantitative polymerase chain reaction (RT-qPCR) analysis (ThermoFisher 4444436) in triplicate for N1, N2, and pepper mild mottle virus (PMMoV) amplicons. The SARS-CoV-2 RNA concentration quantified through the RT-qPCR was normalized using PMMoV concentration in the sample to reduce the noise caused by the dilution, population size, and wastewater flow. The sampling, wastewater process, and RT-qPCR protocols have been extensively validated with a machine-based and manual-aid quality check to ensure the data quality.

To preserve the anonymity of participating utilities and to improve their representativeness, data was aggregated based on county and sample amount [1], as detailed below.

For each sampling location, if there is more than one sample in a week, the concentrations of samples within each week were aggregated using an unweighted average.

For each county, in a certain week, the concentrations obtained from each sampling location within the county were aggregated using a weighted average. The weight for a sampling location is relevant to the sewershed population, or 300,000, whichever is smaller. When a sampling location serves multiple counties, the location is associated with the single county that the wastewater operator has provided as the plant's primary service area.

Text 2 Random forest models

The construction of random forest models in the R package RandomForest, briefly follows the following steps, for a given training set, a subset of the training data (usually 2/3, so-called in-bag) is selected with replacement, while the other 1/3 of the training, so-called out-of-bag (OOB) set, is kept out of training for evaluating the goodness of fit. From each subset of the training dataset (in-bag set) with k variables, a subset of variable m ($m<k$) is randomly selected as a subset to create a decision tree. In an individual decision tree, splitting continues until a maximum tree depth is reached. The final result is obtained by averaging the predictions from all individual trees.

Text 3 Correlation between prediction targets and explanatory factors in case-based and record-based predictions

For case-based predictions, the weekly new cases and the positive rate of the testing showed a moderately strong correlation (R=0.42-0.64) with the all three hospitalization indicators under all four leading times (Fig. S8). Under the same leading time, the weekly new cases and the positive rate showed comparable or slightly stronger correlation with census inpatient sum (Hos_cs, R=0.45-0.64) than census inpatient average (Hos_ca, R=0.44-0.63) and weekly new admission (Hos_wn, R=0.41-0.62). For each hospitalization indicators, the correlation between

the hospitalization indicators and weekly new cases or the positive rate reduced along with the increase of leading time.

For record-based predations, the hospitalization records for each indicator (i.e., Hos_wn, Hos_cs, Hos_ca) in the week of wastewater sampling (Hos0w in the Fig. S8) positively correlated with the future values of these indicators in the next 1-4 weeks (Fig. S8). The correlation between Hos0w and Hos_ca (R=0.53-0.75) was stronger than that of Hos_cs (R=0.46-0.71) and Hos_wn (0.37-0.64) (Fig. S8). The correlation for each indicator also reduced along with the increase of the leading time, with the least correlation coefficient achieved at Hos4w.

Other explanatory factors, including population size, and factors associated with vaccination, CCVI, and the weather showed significant correlations (|R| of 0.01-0.27) with at least one of the targets (Fig. S8). Considering the randomness of random forest algorism, all these explanatory factors were used for establishing case-based or record-based models.

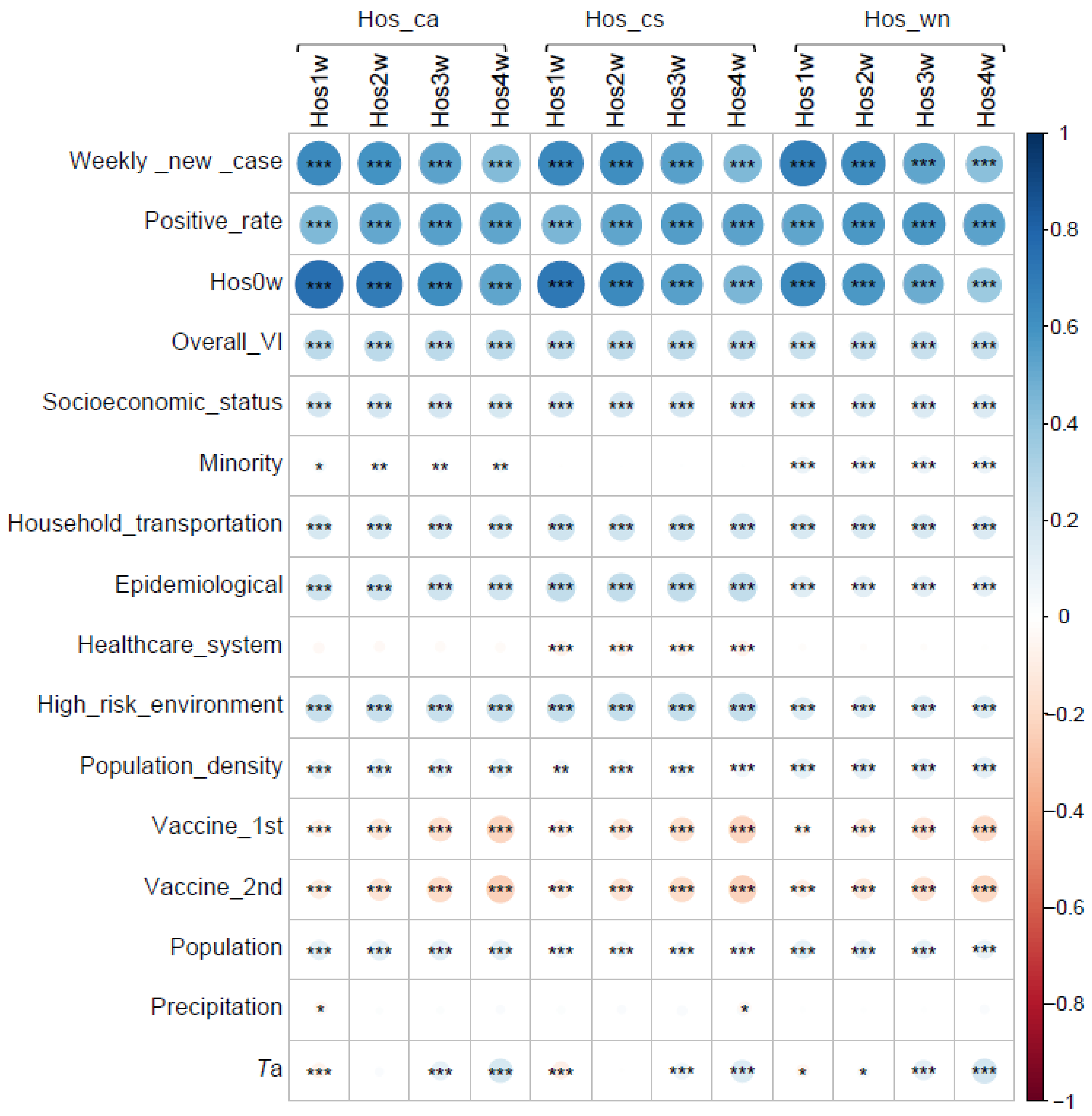

Figure S8. Spearman's correlation between explanatory factors and hospitalization records (three types of hospitalization indicators (i.e. Hos_wn: weekly new admission, Hos_cs: census inpatient sum, and Hos_ca: census inpatient average) in the data used for case-based and record-based models under 4 leading times (Hos1w, Hos2w, Hos3w, Hos4w: the upcoming week, the second, third and fourth week after the wastewater sampling, respectively). Hos0w indicates the hospitalization records for each indicator (i.e., Hos_wn, Hos_cs, Hos_ca) in the week of wastewater sampling. The color and circle size indicate the strength of the correlation (bigger circle=stronger correlation; blue color=positive correlation and red color=negative correlation). The significance of the correlation is determined through two-side t-test, and marked as *, **, and

*** representing a p value of ≥0.01 and <0.05, ≥0.001 and <0.01 and <0.001, respectively. The detailed p values are provided in Table S7.

Reference

1. Duvallet C, *et al.* Nationwide Trends in COVID-19 Cases and SARS-CoV-2 RNA Wastewater Concentrations in the United States. *ACS ES&T Water*, (2022).

2. Hodcroft EB. CoVariants: SARS-CoV-2 Mutations and Variants of Interest. (ed covariants.org) (2021).
