# Supplementary material for: Wastewater-based epidemiology predicts COVID-19-induced weekly new hospital admissions in over 150 USA counties
Source: Nat Commun. 2023 Jul 28;14:4548. doi: 10.1038/s41467-023-40305-x (PMC10382499; doi:10.1038/s41467-023-40305-x)
Supplement: Supplementary file 3 — Reporting Summary [file 41467_2023_40305_MOESM3_ESM.pdf]

nature portfolio

Corresponding author(s): Qilin Wang

Last updated by author(s): Jul 10, 2023

Reporting Summary

Nature Portfolio wishes to improve the reproducibility of the work that we publish. This form provides structure for consistency and transparency in reporting. For further information on Nature Portfolio policies, see our Editorial Policies and the Editorial Policy Checklist.

Statistics

For all statistical analyses, confirm that the following items are present in the figure legend, table legend, main text, or Methods section.

n/a	Confirmed	
☐	☒	The exact sample size (*n*) for each experimental group/condition, given as a discrete number and unit of measurement
☒	☐	A statement on whether measurements were taken from distinct samples or whether the same sample was measured repeatedly
☐	☒	The statistical test(s) used AND whether they are one- or two-sided<br>*Only common tests should be described solely by name; describe more complex techniques in the Methods section.*
☐	☒	A description of all covariates tested
☐	☒	A description of any assumptions or corrections, such as tests of normality and adjustment for multiple comparisons
☐	☒	A full description of the statistical parameters including central tendency (e.g. means) or other basic estimates (e.g. regression coefficient) AND variation (e.g. standard deviation) or associated estimates of uncertainty (e.g. confidence intervals)
☐	☒	For null hypothesis testing, the test statistic (e.g. *F*, *t*, *r*) with confidence intervals, effect sizes, degrees of freedom and *P* value noted<br>*Give P values as exact values whenever suitable.*
☒	☐	For Bayesian analysis, information on the choice of priors and Markov chain Monte Carlo settings
☒	☐	For hierarchical and complex designs, identification of the appropriate level for tests and full reporting of outcomes
☐	☒	Estimates of effect sizes (e.g. Cohen's *d*, Pearson's *r*), indicating how they were calculated

Our web collection on statistics for biologists contains articles on many of the points above.

Software and code

Policy information about availability of computer code

Data collection	No software was used for data collection.
Data analysis	All data analysis was performed using R (version 4.2.0, R Foundation for Statistical Computing, http://www.R-project.org/) with packages involved listed below:<br>For data organization and cleaning: reshape2 (version 1.4.4), dplyr (version 1.0.9), tidyr (version 1.2.2).<br>For model establishment: randomForest (version: 4.7-1.1)<br>For static analysis and model interpretation: frPermute (version 2.5.1), Pdp (version 0.8.1).<br>For data visualization: ggplot2 (version 3.3.6), corrplot (version 0.92), ComplexHeatmap (version 2.15.1), USmap (version 0.6.2).<br><br>The code for data analysis and figures are provided in the link: https://doi.org/10.5281/zenodo.8128697

For manuscripts utilizing custom algorithms or software that are central to the research but not yet described in published literature, software must be made available to editors and reviewers. We strongly encourage code deposition in a community repository (e.g. GitHub). See the Nature Portfolio guidelines for submitting code & software for further information.

Data

Policy information about availability of data

All manuscripts must include a data availability statement. This statement should provide the following information, where applicable:

- Accession codes, unique identifiers, or web links for publicly available datasets
- A description of any restrictions on data availability
- For clinical datasets or third party data, please ensure that the statement adheres to our policy

We used the publicly available data from USA CDC (Centers for Disease Control and Prevention), USA Environmental Protection Agency, and Biobot wastewater surveillance project. The link for each dataset are provided below:
County-level wastewater surveillance data: biobot.io/data
County-level hospitalization data:https://healthdata.gov/Hospital/COVID-19-Reported-Patient-Impact-and-Hospital-Capa/anag-cw7u
County-level COVID-19 Community Vulnerability Index (CCVI) indexes:https://precisionforcovid.org/ccvi
County-level vaccination coverage:https://data.cdc.gov/Vaccinations
Daily county-level COVID-19 cases: https://usafacts.org/visualizations/coronavirus-covid-19-spread-map
Daily temperature and precipitation in each county: Environmental Protection Agency (https://www.ncdc.noaa.gov/cdo-web/datatools/lcd
The data for USA map in figure 2a was sourced from R package 'USmap' where relevant shape data was provided by the US Census bureau (open access): https://data.census.gov/map?layer=VT_2021_040_00_PP_D1&loc=43.3751,-113.1138,z2.6270

The authors are not permitted to share the third party raw data used in the analysis.
Secondary data (wastewater surveillance data and relevant weather, CCVI and hospitalization data) used in the analyses could be shared by contacting the corresponding author (Qilin Wang, Qilin.Wang@uts.edu.au).

Human research participants

Policy information about studies involving human research participants and Sex and Gender in Research.

Reporting on sex and gender	We used the county-level COVID-induced hospitalization numbers without gender information involved (anonymous data).
Population characteristics	The population size in each county were sourced from the 2019 census data from CDC along with vaccination coverage information (https://data.cdc.gov/Vaccinations).<br>The population-related information including the epidemiological factors, socioeconomic status, minority status and language; housing type, transportation, household composition, and disability; epidemiological factors; healthcare system; high-risk environment and population density were based on COVID-19 Community Vulnerability Index (CCVI) from the USA government (publicly available).
Recruitment	N/A
Ethics oversight	Ethical approval was not required for this analysis of anonymous data.

Note that full information on the approval of the study protocol must also be provided in the manuscript.

Field-specific reporting

Please select the one below that is the best fit for your research. If you are not sure, read the appropriate sections before making your selection.

☒ Life sciences ☐ Behavioural & social sciences ☐ Ecological, evolutionary & environmental sciences

For a reference copy of the document with all sections, see nature.com/documents/nr-reporting-summary-flat.pdf

Life sciences study design

All studies must disclose on these points even when the disclosure is negative.

Sample size	Not applicable. The study and analyses were based on observed data that are publicly available. Statistical and modeling analyses were not based on sample size.
Data exclusions	Missing values are excluded from the datasets obtained from the publicly available sources (as the links provided above)
Replication	Not applicable. The study and analyses were based on observed data that are publicly available.
Randomization	Not applicable. The study and analyses were based on observed data that are publicly available.
Blinding	Not applicable. The study and analyses were based on observed data that are publicly available.

Reporting for specific materials, systems and methods

We require information from authors about some types of materials, experimental systems and methods used in many studies. Here, indicate whether each material, system or method listed is relevant to your study. If you are not sure if a list item applies to your research, read the appropriate section before selecting a response.

Materials & experimental systems

n/a	Involved in the study
☒	☐ Antibodies
☒	☐ Eukaryotic cell lines
☒	☐ Palaeontology and archaeology
☒	☐ Animals and other organisms
☒	☐ Clinical data
☒	☐ Dual use research of concern

Methods

n/a	Involved in the study
☒	☐ ChIP-seq
☒	☐ Flow cytometry
☒	☐ MRI-based neuroimaging
